# Combi-Elasto Evaluation of the Degree of Liver Fibrosis in Children with Cholestatic Liver Disease

**DOI:** 10.3390/diagnostics13203229

**Published:** 2023-10-17

**Authors:** Rina Li, Caihui Hu, Fenglin Xu, Qi Zhang, Fazhi Zhou, Chenpeng Zheng, Yang Gao, Yi Tang, Jingyu Chen

**Affiliations:** 1 Department of Ultrasound, Children’s Hospital of Chongqing Medical University, National Clinical Research Center for Child Health and Disorders, Ministry of Education Key Laboratory of Child Development and Disorders, Chongqing Key Laboratory of Pediatrics, Chongqing 400014, China; lirina110@163.com (R.L.); pangfei9264@163.com (C.H.);; 2 FUJIFILM Medical System (Guangzhou) Co., Ltd., Guangzhou 510620, China; 3 Department of Ultrasound, Chongqing Emergency Medical Center, Chongqing 400016, China

**Keywords:** child, cholestatic liver disease, liver fibrosis, combi-elasto

## Abstract

Cholestatic liver disease is a common liver disease in infants and young children. Liver fibrosis is a key factor affecting the prognosis, and liver transplantation is the only treatment option for liver cirrhosis. This study aimed to explore the efficacy of Combi-elasto for diagnosing liver fibrosis in children affected by cholestatic liver disease. A total of 64 children with S1–S4-grade liver fibrosis were enrolled. The general data, routine ultrasound, Combi-elasto, aspartate aminotransferase-to-platelet ratio index (APRI) and Fibrosis-4 (FIB-4) score were compared among children with different grades of liver fibrosis, and the efficacy of the above indexes for evaluating the degree of liver fibrosis was reported. There were remarkable differences in liver size, liver echogenicity, Young’s modulus (E), fibrosis index (FI), activity index (AI) and FIB-4 score among the groups (all *p* < 0.05). E and liver echogenicity were the independent impact factors of liver fibrosis. The areas under the curve of E, APRI, FIB-4 score and the combined model (E+ liver echogenicity) in the evaluation of liver fibrosis were 0.84, 0.61, 0.66 and 0.90, respectively. Ultimately, we concluded that CE is an effective method to evaluate liver fibrosis in children with cholestatic liver disease.

## 1. Introduction

According to statistics, approximately 1 in every 2500 infants is afflicted with cholestatic jaundice, progressing further to cholestatic liver disease [1]. Cholestasis can result from various etiologies, such as infections, anatomical obstructions of the biliary system and genetic and metabolic factors. Among these, biliary atresia (BA) stands out as the predominant cholestatic liver disease in newborns, representing a primary contributor to pediatric liver failure [1,2]. BA has an incidence rate ranging from 1 in 8000 to 18,000 live births, with somewhat elevated prevalence in Asia and Africa compared to Europe. Additionally, it displays a slight predilection for females over males [3]. Left untreated, children with BA frequently die before the age of 2 due to progressive liver fibrosis. Over half of these afflicted children necessitate liver transplantation [4]. Currently, there are no effective pharmacological interventions to prevent or slow the progression of BA in children. Therefore, we need to assess the extent of liver fibrosis in children with cholestatic liver disease as early as possible. This timely assessment serves the purpose of delaying disease progression, reducing reliance on liver transplantation and providing more evidence for clinical decision making when liver transplantation is necessary [5,6].

There are three primary methods for screening liver fibrosis in children with cholestatic liver disease, including liver histopathological biopsy, laboratory test and imaging examination.

Liver histopathological biopsy remains the most accurate diagnostic method to determine the severity of liver fibrosis and cirrhosis [7]. However, it is an invasive procedure with potential sampling errors and is subject to observer variability, making it difficult to perform repeated dynamic observations in clinical practice [8]. Laboratory tests lack tissue specificity and are influenced by various factors, and studies have shown that their effectiveness in assessing liver fibrosis varies [9,10,11,12,13]. The aspartate aminotransferase-to-platelet ratio index (APRI) and Fibrosis-4 (FIB-4) score can be employed to predict the degree of liver fibrosis in individuals with BA [11,12] and to follow up on the prognosis of these patients after Kasai surgery [7,10,13,14]. Imaging examinations, including ultrasound, CT and MRI, are commonly used to diagnose liver diseases [15]. Ultrasound elastography and magnetic resonance elastography (MRE) are the main imaging examinations performed for the detection of liver fibrosis. While MRE has higher diagnostic accuracy, its cost has impeded its widespread use [16]. Ultrasound is the preferred method due to its simplicity, flexibility and lower cost compared to other imaging methods. At present, the advanced elastography techniques applied to evaluate liver fibrosis mainly include real-time tissue elastography (RTE), transient elastography (TE), point shear wave elastography (pSWE) and two-dimensional SWE (2D SWE) [7,17,18,19,20,21]. However, it is imperative to acknowledge that these new ultrasound technologies exhibit specific limitations [7,17,18,19,20,21]. Therefore, there exists an urgent need for a noninvasive, safe, precise and highly repeatable diagnostic method in clinical practice for assessing fibrosis in individuals affected by cholestatic liver disease.

Combi-elasto (CE) combines the advantages of strain elastography and shear wave elastography. It employs the fibrosis index (FI), activity index (AI) and attenuation coefficient (ATT) to accurately assess the levels of fibrosis, inflammation activity and fatty degeneration [22].

Therefore, this study aimed to evaluate the diagnostic value of CE in detecting liver fibrosis by comparing the measurements of CE, including Young’s modulus (E), FI and AI, conventional ultrasound and serum markers. Its goal was to furnish crucial diagnostic insights for fibrosis grading, disease progression prediction and treatment efficacy evaluation, thereby supporting early prevention intervention and improving survival in children affected by cholestatic liver disease.

## 2. Materials and Methods

### 2.1. Study Population

**Figure 1:**
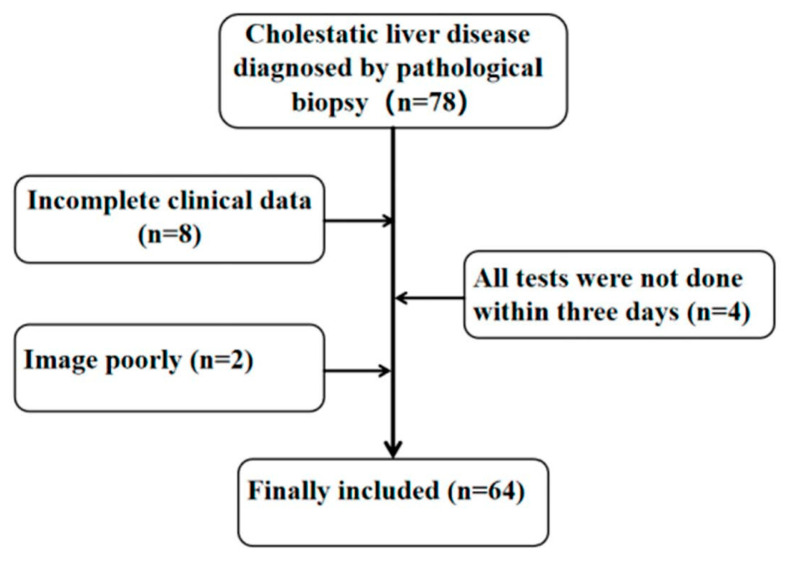
Research object inclusion process.

This study was approved by the Ethics Committee of the Children’s Hospital Affiliated with Chongqing Medical University. We collected cases of children pathologically diagnosed with liver fibrosis caused by cholestatic liver disease at the Affiliated Children’s Hospital of Chongqing Medical University from June 2021 to December 2022. All children completed blood biochemical tests, CE examinations and liver biopsies within three days. The inclusion criteria were as follows. (1) age: 0–18 years old; (2) patients with cholestatic liver disease diagnosed by liver biopsy; and (3) patients without prehepatic ascites. The exclusion criteria were as follows: (1) incomplete data; (2) patients with hepatic space-occupying lesions; (3) patients with other serious systemic diseases, such as heart failure, renal failure and mental illness; and (4) patients after liver transplantation. A flow chart is shown in Figure 1.

### 2.2. Imaging Techniques

The operation was performed by a qualified physician utilizing a ultrasound instrument (Arietta 850; FUJIFILM Healthcare, Tokyo, Japan) and a C252 convex type probe (1–6 MHz, 50 mm radius scan width, 70° field of view scan angle; FUJIFILM Healthcare, Tokyo, Japan). at a frequency of 2–5 MHz. The patient was placed supine, and the left-side position was selected if necessary. The operator placed the probe under the right costal margin or intercostal space to measure the right oblique diameter of the child’s liver and assess its echogenicity. Routine ultrasound evaluation involved two experienced sonographers performing independent assessments of the 2D images. If their evaluations were consistent, the results were recorded. Otherwise, a third sonographer was required to perform a reassessment, and the results were recorded accordingly.

**Figure 2:**
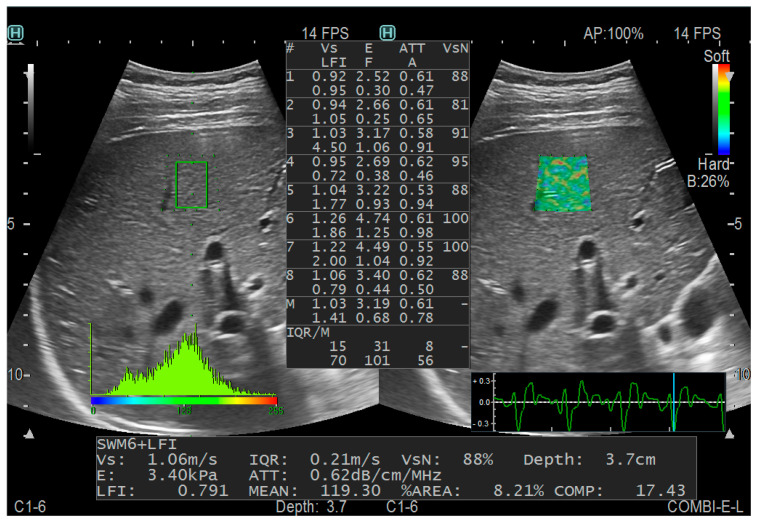
CE measurement diagram.

The measurement was performed in the breath-holding state for children who could assist with breath holding. Those who could not hold their breath were measured in a state of calm breathing. Children who had difficulty cooperating with measurements needed sedative medication. Afterward, the CE mode was selected, and the probe was placed perpendicular to the skin and in the right intercostal space. A sampling frame of dimensions 2.5 cm by 2.5 cm was positioned within the hepatic parenchyma, maintaining a distance of more than 1 cm from the liver capsule and avoiding the large hepatic vessels and the shadow area of the ribs. The E, ATT (to assess the degree of steatosis), FI (to indicate fibrosis) and AI (to indicate inflammation) were measured. To ensure the reliability of the results, the percentage of the net effective shear wave velocity (VsN) was used as the quality control index, which is the percentage of effectiveness. Each patient was measured five times, and the VsN of each measurement was ≥80%. Figure 2 shows a schematic diagram.

### 2.3. Laboratory Indexes

The liver function indexes included aspartate aminotransferase, alanine aminotransferase and total bilirubin. The inflammatory markers included the following: platelet count, APRI and FIB-4 score.

### 2.4. Pathological Diagnoses

All cases included in this study underwent intraoperative liver histopathological biopsy or ultrasound-guided liver biopsy. The site of ultrasound-guided biopsy was chosen to be in basic accordance with the region of interest (ROI) selected for CE examination. Liver fibrosis grading was performed based on the modified Scheuer scoring system, and pathological diagnosis was conducted by the same deputy chief physician in the pathology department with 20 years of working experience [23] (Table 1).

### 2.5. Statistical Analyses

Statistical analysis was performed using SPSS 25.0 software. Count data that conform to normality and homogeneity of variance are represented by $\;\bar{x}$ ± s. Comparisons between two groups was performed using t tests. The median (upper and lower quartiles) represents nonpositive count data with a normal distribution or heterogeneous variance. The Mann-Whitney U test was conducted to compare groups. Kendall’s tau-b correlation coefficient was utilized to evaluate the correlation between fibrosis staging and clinical indicators. When r < 0.2, there was no significant correlation, 0.2 ≤ r < 0.5 indicated a low correlation, 0.5 ≤ r < 0.75 indicated a moderate correlation and r ≥ 0.75 indicated a high correlation. Multiple logistic regression was used to analyze the factors affecting the grading of liver fibrosis. Receiver operating characteristic (ROC) curves were generated to evaluate parameters showing significant correlations with liver fibrosis stage. The analysis aimed to determine the optimal cutoff values, sensitivity, specificity, positive predictive value (PPV), negative predictive value (NPV) and the areas under the ROC curve (AUCs) for distinguishing between fibrosis and cirrhosis. *p* < 0.05 was considered statistically significant.

## 3. Results

### 3.1. General Characteristics

Our study comprised 64 patients, including 33 males and 31 females, aged between 1 month and 4 years, with a median age of 0.21 years. Among these, 56 children received intraoperative liver histopathological biopsies, while 8 children underwent ultrasound-guided percutaneous liver biopsies. The grading of liver fibrosis in the children was distributed as follows: 9 cases at stage S1, 11 cases at stage S2, 18 cases at stage S3 and 26 cases at stage S4. They were categorized into two groups based on the extent of liver fibrosis. The clinical information of all patients and comparisons between the fibrosis and cirrhosis groups are shown in Table 2. There was no statistical significance in age, sex or BMI (*p* > 0.05).

### 3.2. Comparative Study of Conventional Ultrasound and CE

There were significant differences in liver echo, liver size and CE value (E, FI, AI) among patients with different liver fibrosis grades (*p* < 0.01). E, FI and AI all showed an increasing trend with increasing fibrosis degree.

The correlation analysis indicated that liver size, liver echo and CE measurements exhibited positive correlations with the extent of liver fibrosis, among which E was moderately correlated with the histological grade of liver biopsy, with a correlation coefficient of 0.542 (*p* < 0.01), while the remaining variables showed weaker correlations.

**Figure 3:**
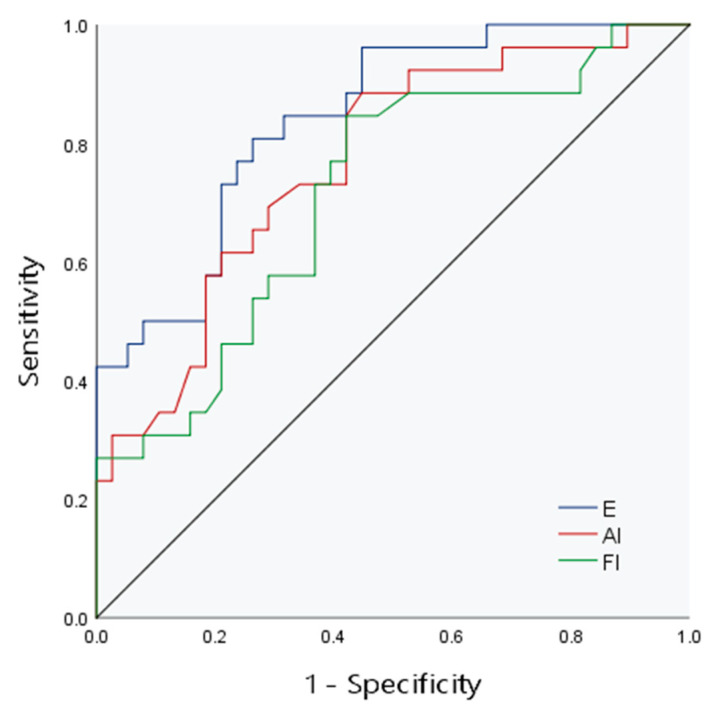
The ROC curve of CE for evaluation of liver fibrosis.

**Figure 4:**
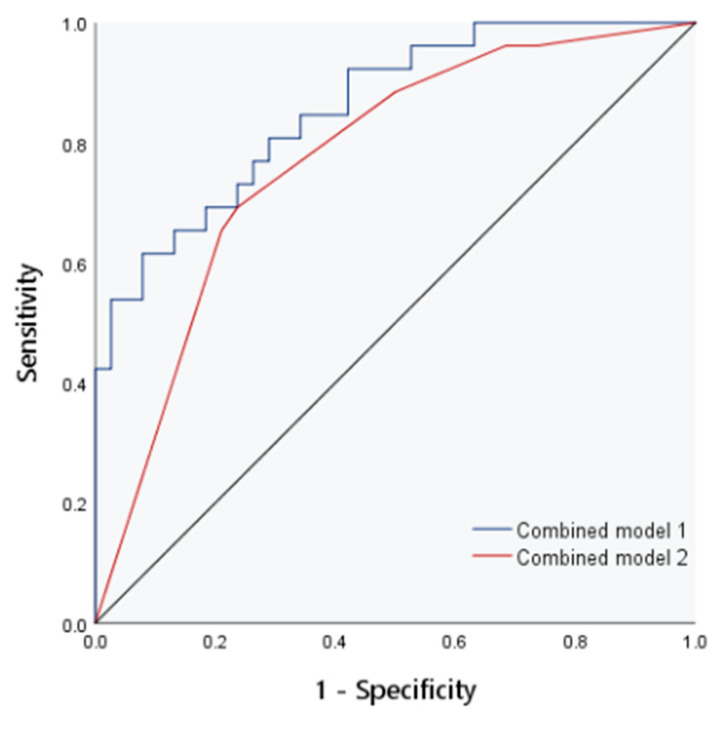
The ROC curve of combined models 1 and 2 for evaluation of liver fibrosis.

The AUCs of E, FI and AI to evaluate the degree of liver fibrosis in children were 0.84, 0.72 and 0.77, respectively. In the collinearity diagnosis among independent variables, collinearity between FI and AI was found. To ensure the reliability of the model results, one variable had to be eliminated, so AI was eliminated according to the clinical significance. Combined model 1 was composed of E and FI to evaluate the diagnostic efficacy of CE, and the AUC was 0.86. Combined model 2 combined liver echo and liver size to evaluate the diagnostic efficacy of conventional ultrasound, and the AUC was 0.77 (Figure 3 and Figure 4, Table 3)

### 3.3. Diagnostic Efficacy of Laboratory Indexes

**Figure 5:**
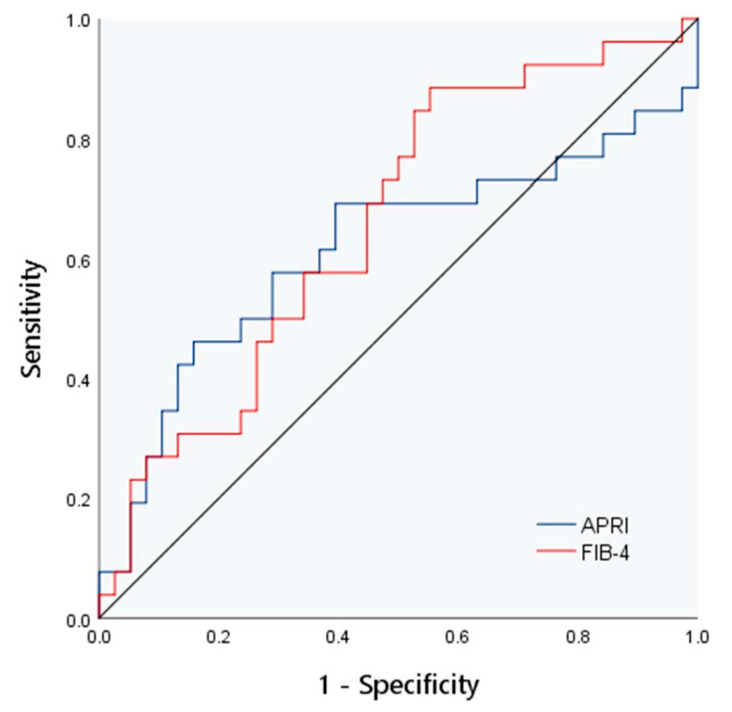
The ROC curve of APRI and FIB-4 for evaluation of liver fibrosis.

There were significant differences in FIB-4 among the different hepatic fibrosis groups (*p* < 0.05). The correlation analysis showed that all laboratory indexes and models had no association with liver fibrosis grade (*p* > 0.05). The areas under the ROC curves of the APRI and FIB-4 index for evaluating liver fibrosis in children were 0.61 and 0.66, respectively, as shown in Figure 5.

### 3.4. Multivariate Analysis of Grading of Hepatic Fibrosis in Children with Cholestatic Liver Disease

**Figure 6:**
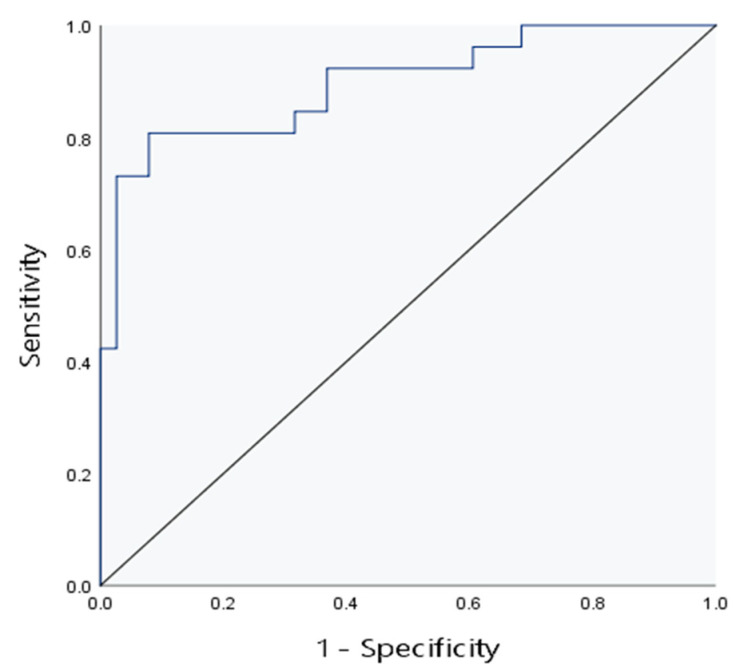
The ROC curve of combined model 3 for evaluation of liver fibrosis.

To determine the impact of various factors on hepatic fibrosis in children with cholestatic liver disease, hepatic fibrosis was taken as the dependent variable, and E, FI, liver echogenicity, liver size and AST were taken as independent variables. Then, binary logistic regression analysis was adopted. The analysis results are presented in Table 4. As shown in the table, E and liver echogenicity were selected as independent predictors with statistical significance. To further improve the diagnostic efficiency of liver fibrosis in children with cholestatic liver disease, two independent influencing factors (E+ liver echo) were used to construct combined model 3. The AUC in the evaluation of liver fibrosis was 0.90. The positive predictive value and negative predictive value of combined model 3 for predicting liver fibrosis in children are both 87.5%, higher than the other four models. See Figure 6 and Table 5.

## 4. Discussion

Cholestatic liver diseases, including BA, are the main cause of liver fibrosis in early infancy and the leading cause of liver transplantation in children [5]. Fibrosis can determine the quality of life and prognosis of patients with cholestatic liver diseases [24]. Despite the emergence of new strategies for treating liver fibrosis proposed by scholars [25,26], there is still a lack of clear clinical trials to validate their efficacy. Accordingly, early detection and delaying the progression of liver fibrosis remain the most effective approaches to enhance the prognosis of children afflicted with cholestatic liver diseases.

With the advancement of ultrasound elastography, ultrasound can not only provide essential information on liver morphology and hemodynamics but also measure the physical properties of liver hardness or elastic modulus. Thus, ultrasound may become one of the most important means of noninvasive evaluation for liver lesions. At present, the elastic imaging techniques performed to evaluate the degree of liver fibrosis mainly include RTE, TE and SWE. Naganuma et al. [27] demonstrated that SWE exhibits a remarkably larger area under the ROC curve than RTE in diagnosing liver fibrosis. However, SWE measurements can be easily influenced by the degree of liver inflammation [19]. In contrast, RTE remains unaffected by the degree of inflammation and primarily mirrors the extent of liver fibrosis [28]. 

CE is an emerging ultrasound elastography technique that combines the technical advantages of shear wave elastography and strain elastography. It has evolved into a comprehensive quantitative analysis technique, encompassing multiple factors and parameters [22]. In this study, we compared the diagnostic and clinical values of CE, routine ultrasound and indirect serum markers for evaluating liver fibrosis. The cutoff values of E, FI, AI, APRI and FIB-4 were 13.06 KPa (Sen: 80.80%, Spe: 73.70%), 2.01 (Sen: 84.60%, Spe: 57.90%), 1.37 (Sen: 88.50%, Spe: 55.30%), 1.59 (Sen: 46.20%, Spe: 84.20%) and 0.01 (Sen: 88.50%, Spe: 44.70%), respectively. The AUCs of CE, APRI and FIB-4 were 0.86, 0.61 and 0.66, respectively. The combined model had a higher positive predictive value than the single index.

Currently, there is no research available, both domestically and internationally, regarding liver stiffness measurement in children using CE. Therefore, we can only refer to the research data from other elastic imaging techniques. Studies have shown that combining strain elastography and shear wave imaging can enhance the diagnostic accuracy of liver fibrosis and inflammation [29,30]. A meta-analysis evaluated the diagnostic value of elastography in the assessment of fibrosis stages in cholestatic liver diseases. Natalia et al. [31] indicated that TE had a sensitivity and specificity of 71% and 93%, respectively. Another study demonstrated that elastography differentiated stage F4 fibrosis from F0–F3 fibrosis with a sensitivity of 96% and specificity of 89% [32]. Additionally, researchers have compared the diagnostic performance of 2D SWE in assessing liver fibrosis in pediatric patients with autoimmune hepatitis, BA and other chronic liver diseases. The study indicated that the cutoffs for predicting advanced fibrosis (≥F3) were 16 kPa (AUC: 0.98; sensitivity: 87.5%; specificity: 96.7%) in BA and 8.7 kPa (AUC: 0.98; sensitivity: 93.8%; specificity: 96.1%) in other chronic liver diseases [33]. A systematic review reported that SWE was an advanced ultrasonography technique that had a good pooled diagnostic performance (sensitivity: 83%; specificity: 77%; AUC: 0.896) for liver fibrosis due to BA [34]. An AUC exceeding 0.8 is commonly regarded as a strong indicator of diagnostic performance [35]. Therefore, CE also has a high diagnostic efficacy for liver fibrosis.

APRI and FIB-4 can indirectly reflect the degree of liver fibrosis. Currently, they are applied to the study of liver disease in children, such as cystic fibrosis liver disease [36], chronic viral hepatitis [37], non-alcoholic fatty liver disease [38] and BA. A study showed that FIB-4 failed to correlate with the fibrosis stage, and the AUC of APRI was 0.56 for cirrhosis in children with BA [39]. According to another report, with an APRI of 1.38, the sensitivity and specificity to detect fibrosis/cirrhosis were 100% and 21.43%, respectively; thus, APRI is ineffective in clinical practice for assessing fibrosis or cirrhosis in patients with non-BA cholestasis [40]. Similar to our study findings, APRI and FIB-4 have a poor diagnostic efficacy in children with cholestatic liver disease.

The factors related to fibrosis grading in multiple-factor logistic regression analysis (E+ liver echo) were formed into combined model 3. The diagnostic efficacy of the above model for liver fibrosis was higher than that of a single diagnostic method. In summary, CE can effectively evaluate the degree of liver fibrosis in children. Combined conventional ultrasound can improve its efficiency.

The present study has several limitations. First, it is the first study to apply CE to children with cholestatic liver disease, and all study results only apply to the Combi-elasto mode of the Hitachi Arietta 850. Second, this is a single-center study, so prospective studies in multiple cohorts are required to verify the present results. Finally, the biological mechanism of CE is unclear. Molecular mechanism research and large-sample multi-center studies of CE remain to be conducted in the future.

## 5. Conclusions

We conclude that CE is an effective tool for evaluating liver fibrosis in children with cholestatic liver disease, especially for distinguishing between hepatic fibrosis and cirrhosis. It has higher diagnostic efficacy than APRI and FIB-4 score. Moreover, combining CE with conventional ultrasound can improve the diagnostic performance. 

## Figures and Tables

**Table 1:** Modified Scheuer scoring system.

1. Fibrosis grade	2. Optical characteristics of liver tissue
3. S0	4. Normal
5. S1	6. Liver portal area enlarged and fibrosis
7. S2	8. Fibrous septum formed and lobular structure was retained
9. S3	10. Lobular structure disturbance, no cirrhosis
11. S4	12. Cirrhosis

**Table 2:** Clinical information of all patients and comparisons between liver fibrosis and liver cirrhosis group according to the histological findings.

	All Children (*n* = 64)	Liver Fibrosis (F1-2-3) (*n* = 38)	Liver Cirrhosis (F4) (*n* = 26)	*p*
Age, year	0.21 (0.17, 0.50)	0.17 (0.15, 0.44)	0.38 (0.17, 0.87)	0.213
Sex, *n* (%)				
Male	33 (51.56)	17 (44.74)	16 (61.54)	0.190
Female	31 (48.44)	21 (55.26)	10 (38.46)	
BMI, kg/m^2^	15.60 ± 2.17	15.57 ± 2.36	15.66 ± 1.89	0.870
Conventional ultrasound echo, *n* (%)				
Medium homogeneous	20 (31.25)	17 (44.74)	3 (11.54)	<0.001
Enhanced homogeneous	17 (26.56)	12 (31.58)	5 (19.23)	
Enhanced inhomogeneous	27 (42.19)	9 (23.68)	18 (69.23)	
liver enlargement, *n* (%)	49 (76.56)	25 (65.79)	24 (92.31)	0.015
E, kPa	12.67 (9.19, 16.62)	10.53 (7.29, 13.33)	16.09 (13.37, 27.04)	<0.001
ATT, dB/cm/MHz	0.62 ± 0.11	0.62 ± 0.10	0.63 ± 0.12	0.723
FI	2.41 ± 0.94	2.11 ± 0.74	2.85 ± 1.03	0.001
AI	1.49 ± 0.37	1.35 ± 0.30	1.70 ± 0.37	<0.001
AST, U/L	104.00 (47.25, 227.75)	101.00 (47.00, 183.75)	217.50 (45.25, 307.25)	0.127
ALT, U/L	104.50 (37.25, 152.00)	99.00 (37.75, 146.75)	106.50 (30.25, 165.00)	0.940
T.bil, µmol/L	142.35 (14.55, 191.33)	76.55 (15.30, 176.63)	154.00 (12.00, 213.48)	0.547
PLT, ×10^9^/L	350.66 ± 147.07	366.82 ± 158.18	327.04 ± 128.45	0.292
APRI	0.75 (0.48, 1.79)	0.63 (0.47, 1.11)	1.10 (0.44, 2.63)	0.122
FIB-4	0.07 (0.04, 0.38)	0.06 (0.03, 0.29)	0.14 (0.06, 0.61)	0.033

Abbreviations: BMI: body mass index, E: Young’s modulus, ATT: attenuation coefficient, FI: fibrosis index, AI: activity index, AST: aspartate transaminase, ALT: alanine aminotransferase, TBil: total bilirubin, PLT: platelet, APRI: aspartate aminotransferase-to-platelet ratio index, FIB-4: Fibrosis-4.

**Table 3:** Efficacy of CE indexes in evaluating liver fibrosis in children.

Variables	Cutoff Value	AUC	95% CI	*p*	Sensitivity, %	Specificity, %	Youden Index	PPV, %	NPV, %
E	13.06 kPa	0.84	0.75–0.94	<0.001	80.80	73.70	0.55	67.74	84.85
FI	2.01	0.72	0.59–0.84	0.004	84.60	57.90	0.43	57.89	84.62
AI	1.37	0.77	0.65–0.88	<0.001	88.50	55.30	0.55	57.50	87.50

Abbreviations: AUC: area under the curve, CI: confidence interval, PPV: positive predictive value, NPV: negative predictive value.

**Table 4:** Multivariate analysis of liver fibrosis in children with cholestatic liver disease.

Variables	*β*	SE	OR	95% CI	*p*
E	0.436	0.156	1.546	1.138–2.100	0.005 *
FI	−0.994	0.966	0.370	0.056–2.456	0.303
Liver echo	2.060	1.034	7.847	1.034–59.549	0.030 *
Liver size	−0.177	1.102	0.838	0.097–7.262	0.872
AST	0.001	0.003	1.001	0.996–1.007	0.594

* *p* < 0.05, Abbreviation: SE: standard error.

**Table 5:** Combined model 1–5 evaluated the efficacy of different liver fibrosis grades.

Variables	Cutoff Value	AUC	95% CI	*p*	Sensitivity, %	Specificity, %	Youden Index	PPV, %	NPV, %
Combined model 1	-	0.86	0.77–0.95	<0.001	61.50	92.10	0.54	84.21	77.78
Combined model 2	-	0.77	0.66–0.89	<0.001	69.20	76.30	0.46	66.67	78.38
Combined model 3	-	0.90	0.81–0.98	<0.001	80.80	92.10	0.73	87.50	87.50
FIB-4	0.01	0.66	0.52–0.79	0.033	88.50	44.70	0.33	52.27	85.00
APRI	1.59	0.61	0.46–0.77	0.122	46.20	84.20	0.30	66.67	69.57

## Data Availability

Data are unavailable due to privacy or ethical restrictions.
